# Comparing Small and Large Genomes Within Monogonont Rotifers

**DOI:** 10.1093/gbe/evaf041

**Published:** 2025-03-06

**Authors:** Jonathon E Mohl, Patrick D Brown, Aaron J Robbins, Philip Lavretsky, Rick Hochberg, Robert L Wallace, Elizabeth J Walsh

**Affiliations:** Department of Mathematical Sciences, University of Texas at El Paso, El Paso, TX 79968, USA; Department of Biological Sciences, University of Texas at El Paso, El Paso, TX 79968, USA; Department of Biological Sciences, University of Texas at El Paso, El Paso, TX 79968, USA; Department of Biological Sciences, University of Texas at El Paso, El Paso, TX 79968, USA; Department of Biological Sciences, University of Massachusetts Lowell, Lowell, MA 01854, USA; Department of Biology, Ripon College, Ripon, WI 54971, USA; Department of Biological Sciences, University of Texas at El Paso, El Paso, TX 79968, USA

**Keywords:** body size, genome size, metabolic rate, repetitive elements, Rotifera

## Abstract

Genome size is an important correlate of many biological features including body size, metabolic rate, and developmental rate and can vary due to a variety of mechanisms, including incorporation of repetitive elements, duplication events, or reduction due to selective constraints. Our ability to understand the causes of genome size variation is hampered by limited sampling of many nonmodel taxa, including monogonont rotifers. Here, we used high-throughput Nanopore sequencing and flow cytometry to estimate genome sizes of nine species of monogonont rotifers representing seven families, including three representatives of Superorder Gnesiotrocha. We annotated the genomes and classified the repetitive elements. We also compared genome size with two biological features: body size and metabolic rate. Body sizes were obtained from the literature and our estimates. Oxygen consumption was used as a proxy for metabolic rate and was determined using a respirometer. We obtained similar genome size estimates from genome assemblies and flow cytometry, which were positively correlated with body size and size-specific respiration rate. Importantly, we determined that genome size variation is not due to increased numbers of repetitive elements or large regions of duplication. Instead, we observed higher numbers of predicted proteins as genome size increased, but currently many have no known function. Our results substantially expand the taxonomic scope of available genomes for Rotifera and provide opportunities for addressing genetic mechanisms underlying evolutionary and ecological processes in the phylum.

## Graphical Abstract

**Graphical Abstract:**
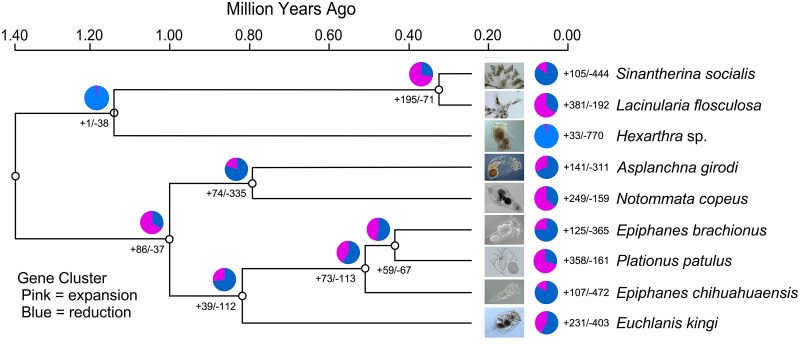

**Significance**

We provide nine new monogonont rotifer genomes, substantially extending the phylogenetic range from Brachionidae and Proalidae to include six additional families. We compared two approaches to estimate genome size to determine consistency in size estimates based on sequencing and flow cytometry. Using these estimates, we investigated potential mechanisms for genome expansion (occurrence and types of repetitive elements). We found a positive relationship between genome size, body size, and size-specific metabolic rate.

## Introduction

Genome size differences among lineages, the biological outcomes of genome content, and the mechanisms that cause expansion or reduction underlying these patterns are all components of the *C*-value enigma. The biological foundation for these relationships is an ongoing topic of debate (Elliott and Gregory 2015). Many factors determine genome size including physiological traits such as cell size (i.e. the *C*-value enigma; Elliott and Gregory 2015), development time (Gregory 2002), and metabolic rate (Hughes and Hughes 1995; Gregory 2005; Wright et al. 2014). In particular, the relationship between genome size and metabolism is generally inverse, with higher metabolic rates corresponding to smaller genome sizes, smaller cell sizes, and faster life cycles (Hughes and Hughes 1995; Gregory 2005; Wright et al. 2014). In addition, features of the genome itself can contribute to increased size such as polyploidization, gene duplication, repetitive elements, and high rates of horizontal gene transfer and acquisition of transposable elements (TEs) (Gregory 2005; Lynch 2007; Canapa et al. 2015). Moreover, genome sizes may be phylogenetically constrained (e.g. Alfsnes et al. 2017). Finally, ecological features such as effective population sizes can also drive genome size evolution where purifying selection can remove excess DNA (Lynch and Conery 2003), or segmental deletions can result in smaller genomes as can the loss of TEs (Kapusta et al. 2017).

Rotifers are small, ubiquitous, aquatic invertebrates that are eutelic, whereby they increase body size by cell expansion rather than by cell number (Birky and Field 1966; Pagani et al. 1993; Wallace 2002). Although a relatively small taxonomic group, they exhibit a diversity of life cycles and lifestyles. Traditionally, they are composed of three major groups with ∼2,000 morphospecies in 36 families: the obligately sexual Seisonacea, the obligately asexual Bdelloidea, and the cyclical parthenogenetic Monogononta. Within the monogononts, Superorder Gnesiotrocha includes species with diverse lifestyles including those that are holoplanktonic and those that are sessile and/or colonial (Wallace et al. 2015). Importantly, being eutelic organisms, changes in cell size may influence body size and potentially link genome size with physiological processes as genome size is typically negatively correlated with metabolism (Gregory 2001, 2005). Conversely, genome size may be decoupled from cell size, and thus, metabolism in eutelic animals.

To date, many studies that have attempted to understand genome heterogeneity of rotifers determined genome size via flow cytometry, which includes eight bdelloids (e.g. Pagani et al. 1993; Mark Welch and Meselson 1998, 2003) and 43 monogonont rotifers, mostly from the genus *Brachionus* (e.g. Mark Welch and Meselson 1998; Stelzer 2011; Stelzer et al. 2011; Brown and Walsh 2019). In general, genome sizes have ranged from 0.05 pg for *Hexarthra mira*, *Hexarthra fennica*, and *Hexarthra* sp. to 0.42 pg in some populations of *Brachionus asplanchnoidis* (Stelzer et al. 2011; Riss et al. 2017; Brown and Walsh 2019). Interestingly, genome size estimates based on whole-genome sequencing in bdelloid rotifers seem to be generally lower than those obtained from fluorometric methods (Mark Welch and Meselson 2003; Flot et al. 2013) and likely are attributable to the presence of heterochromatin and repeated regions (Bennett et al. 2003; Nishibuchi and Déjardin 2017). However, few studies have explored the mechanisms involved in genome expansion or contraction in rotifers. Walsh and Zhang (1992) reported that triploid individuals of the monogonont rotifer *Euchlanis dilatata* are significantly larger than their diploid counterparts. Similarly, in *B. asplanchnoidis* genome size has a positive linear relationship with body size, egg size, and egg development time (Stelzer et al. 2021). Blommaert et al. (2019) found that repetitive elements account for most genome size differences in the *Brachionus plicatilis* species complex (44% in *B. asplanchnoidis*, 14% to 25% in other members of the complex). Conversely, Stelzer et al. (2023) noted that genome size streamlining appears to be overridden by genotypic differences when adapting to a new environment.

Here, we report newly sequenced monogonont rotifer genomes representing seven families, eight genera, and nine species (*Asplanchna girodi*, *Plationus patulus*, *Epiphanes brachionus*, *Epiphanes chihuahuaensis*, *Euchlanis kingi*, *Lacinularia flosculosa*, *Notommata copeus*, *Hexarthra* sp., and *Sinantherina socialis*) (Table 1). Genome sizes were estimated by both whole-genome sequencing and flow cytometry. Protein-coding regions and repetitive elements were identified and classified to determine their roles in genome size expansion. Finally, we describe features of the genomes to determine correlates with physiological processes such as body size and metabolism. Given the variety of causes for genome size discrepancies, we posit that genome size will be positively related to body size and respiration rates.

**Table 1:** ONT sequencing results for nine monogonont rotifer species representing seven families

Species	Reads	Base pairs (Gb)	ONT read N50
*Asplanchna girodi*	4,517,325	14.2	4,586
*Euchlanis kingi*	5,411,827	5.6	1,673
*Epiphanes brachionus*	4,633,325	6.0	1,521
*Epiphanes chihuahuaensis*	7,558,521	7.1	1,341
*Lacinularia flosculosa*	7,355,737	16.7	3,616
*Notommata copeus*	12,201,471	15.6	2,798
*Plationus patulus*	9,278,337	18.5	3,743
*Hexarthra* sp.	8,617,281	12.3	2,834
*Sinantherina socialis*	1,170,649	4.2	4,974

## Results

### Sequencing and Genome Assembly

After the extraction and amplification of the genomic DNA, results from a single sequencing run using an Oxford Nanopore MinION MK1C per sample are summarized in Table 1 and supplementary document S1, Supplementary Material online. Briefly, the number of reads ranged from 1.2 to 12.2 million reads per sample. The total number of bases ranged from 4.2 to 18.5 billion. The N50 of the Oxford Nanopore Technology (ONT) reads ranged from 1,341 to 4,974.

The assembled scaffolds contained between 260 (*Hexarthra* sp.) and 9,684 (*P. patulus*) scaffolds after filtering for contigs > 1,000 bases long (Table 2; supplementary document S1, Supplementary Material online) and removing contamination and potential duplications due to assembly errors. The longest scaffold was within the *Hexarthra* sp. assembly and was 4.58 million bases long. The genome assembly N50 values ranged from 51,793 to 2,064,958, and the coverage ranged from 39× to 320×. The GC content in the assembled genomes ranged from 24.95% to 31.14%, with the larger genomes having a lower GC content compared to the smaller genomes. Finally, correlations between the size of the genomes estimated from genome assembly and flow cytometry were positive (*R*^2^ = 0.83, *P*-value: 0.004).

**Table 2:** Assembled genome characteristics for selected monogonont rotifer species

Species	Total length of contigs (bp)	Scaffolds	Largest	N50	L50	GC%	Coverage (X)	Busco—complete	Proteins
*Asplanchna girodi*	140,467,571	4,455	524,610	71,869	549	27.62	101.1	83.9	22,211
*Euchlanis kingi*	122,941,129	3,585	2,101,693	146,227	187	26.45	45.27	93.3	28,646
*Epiphanes brachionus*	100,452,093	4,073	359,666	51,793	544	25.54	59.31	89.0	21,474
*Epiphanes chihuahuaensis*	71,425,459	2,959	351,612	63,931	324	29.88	99.38	88.6	18,205
*Lacinularia flosculosa*	139,982,605	4,200	1,270,754	108,456	315	25.88	119.38	95.3	31,550
*Notommata copeus*	195,270,668	7,511	996,338	88,310	472	25.7	79.89	91.4	33,943
*Plationus patulus*	216,357,354	9,684	714,854	65,640	804	24.95	85.53	93.3	36,604
*Hexarthra* sp.	38,212,941	260	4,583,065	2,064,958	7	31.03	314.40	96.5	11,093
*Sinantherina socialis*	107,055,129	1,652	2,927,615	283,838	94	25.04	39.24	85.5	22,563
*Proales similis*	32,722,929	15	3,837,811	2,428,301	6	45.51	ND	95.7	14,641
*Brachionus plicatilis*	106,939,251	716	6,584,681	1,156,906	29	26.75	ND	92.5	19,586

Values for *B. plicatilis* and *P. similis* were taken from Franch-Gras et al. (2018) and Kim et al. (2021), respectively. The *P. similis* genome was determined using PacBio sequel sequencing.

ND, not determined.

### Repetitive Elements

**Fig. 1.**
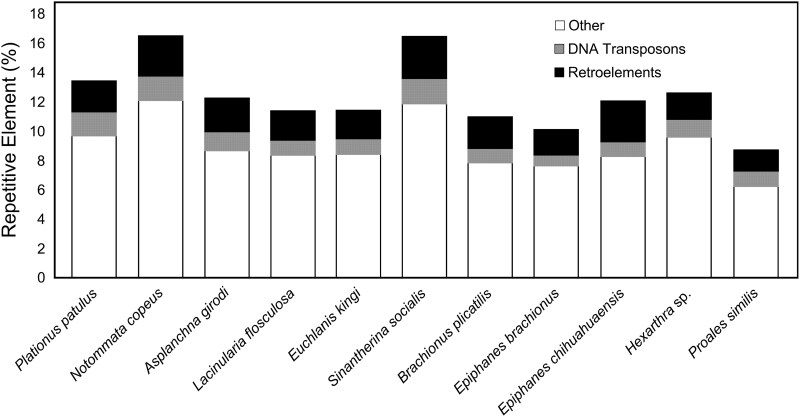
Genomic percentages of various repetitive elements found by RepeatMasker for the assembled rotifer genomes. Assembled genomes are sorted from the largest to the smallest. Black = retroelements, gray = DNA transposons, and white = other repetitive elements. See supplementary document S1, Supplementary Material online for complete results.

Repetitive elements were categorized into retroelements (e.g. SINEs, LINES, and LTR elements), DNA transposons (e.g. hobo-Activator and Tc1-15630-Pogo), and “other” (e.g. rolling circles, simple repeats, satellites, and small RNAs). Percentages of repetitive elements were determined based on the Metazoan library (Fig. 1; see supplementary document S1, Supplementary Material online for full results of RepeatMasker analyses). The overall percentages of repetitive elements in sequenced genomes ranged from 9% to 17% in *Proales similis* and *N. copeus*, respectively. Of note, *N. copeus* had the largest percentage in the “other” category, which was driven by an increase in simple repeats and total interspersed repeats of 4.79% and 4.83%, respectively, while the *P. patulus* genome, the second largest, had 3.33% and 4.15%. The two smallest assemblies, *Hexarthra* sp. and *P. similis*, had the highest percentage of unclassified repeats of 1.59% and 1.35%, respectively. The species with the next highest unclassified percentage was *N. copeus* at 0.84%. The number of repetitive elements was linearly related to the size of the nonrepetitive DNA (nonrepeat DNA: pg = 4.99, repetitive DNA: pg = 0.0268; *R*^2^ = 0.88, *P*-value: 1.84 × 10^−5^). Using SyMAP, no large, duplicated regions were identified within these genomes (results not shown).

### Predicted Protein Comparisons

Using Braker2, the number of predicted proteins found ranged from 11,093 to 36,604. BUSCO completeness scores, based on the predicted proteins, ranged from 83.9% to 96.5% across the genomes. The extremes in these values at the low end were found in *Hexarthra* (smallest assembled genome) and at the high end in *P. patulus* (largest genome) (Table 2). To show that a majority of proteins were on larger contigs and not potential duplications on the smaller contigs, we determined the percentage of contigs in which 90% of the proteins were found. These ranged from 8% of the *Hexarthra* contigs to 63% for *E. brachionus* (see supplementary document S3, Supplementary Material online for additional details). The number of contigs and protein numbers were also positively correlated (*R*^2^ = 0.73, *P*-value: 0.003; supplementary document S1, Supplementary Material online).

**Fig. 2.**
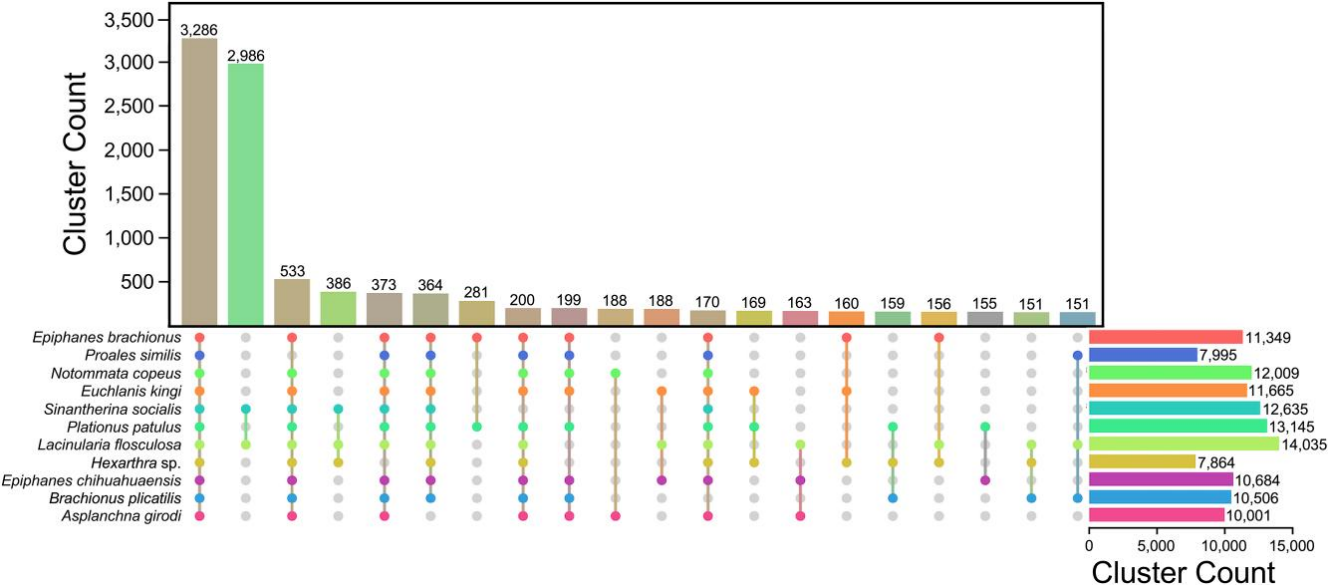
OrthoVenn3 comparison of shared proteins among rotifer species. The bar chart shows the number of shared protein clusters among different species sets. Each colored sphere indicates the species associated with the shared gene clusters.

OrthoVenn3 analysis showed a total of 29,087 protein clusters. There were 3,286 clusters, which included 46,093 proteins that were shared across the 11 genomes (see Fig. 2; supplementary document S2, Supplementary Material online). Additionally, of those clusters, 1,284 contained a single protein copy from each genome. The two colonial gnesiotrochan rotifers, *L. flosculosa* and *S. socialis*, shared 2,986 protein clusters (9,558 proteins) specific to the two species making it the second largest overlap. Comparing the remaining overlapping groups, there are similar numbers of proteins found from each assembly (supplementary document S2, Supplementary Material online). Singletons for each of the genomes ranged from 202 to 7,223 proteins in *Hexarthra* and *N. copeus*, respectively.

**Fig. 3.**
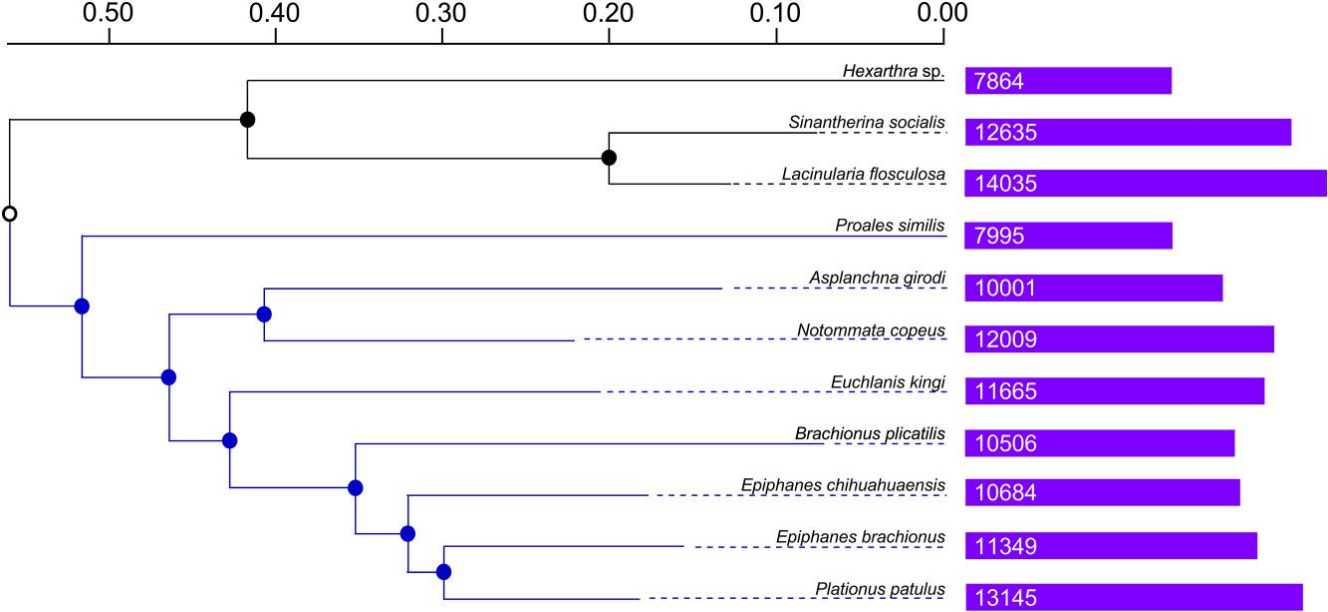
OrthoVenn3 neighboring joining phylogenetic analysis of rotifer species based on identified protein clusters. The upper three branches (in black) branches are species from Superorder Gnesiotrocha, and the remaining branches (in blue) are from Superorder Pseudotrocha.

Using the protein clusters of each assembled genome, a phylogenetic analysis was conducted in OrthoVenn3 (Fig. 3). Gnesiotrocha formed a separate group from that of Superorder Pseudotrocha, supporting traditional phylogenetic separation of these two superorders. In both cases, the assembled genomes that are the smallest appear as outgroups within the respective superorders. The expansion and contraction of the protein clusters across the various taxa are depicted in supplementary document S3, Supplementary Material online.

### Metabolic Rate and Genome Size

Rotifer body lengths without the foot ranged from 88 to 1,081 (µm) (Table 4). Genome size was positively correlated with size-specific respiration rate in rotifers (coefficient: 4.30, *P*-value: 0.02, marginal *R*^2^: 0.49, conditional *R*^2^: 0.94), contrary to the predictions of the metabolic theory of genome size.

## Discussion

Prior to this study, genome size estimates for monogonont rotifers were mostly from the family Brachionidae, limiting our understanding of genome evolution within the Rotifera and metazoans in general. Additionally, genome sizes have been estimated using two methods (flow cytometry and direct sequencing), and the correspondence between the methods was understudied for nonmodel taxa, confounding direct comparisons among genomes. We sequenced the genomes of nine rotifer species from seven monogonont families and measured their genome sizes via flow cytometry, thus substantially increasing the phylogenetic breadth of genomic sizes and content estimates. We found that values obtained by the two methods were roughly comparable and, in general, genome sizes found in this study are within the range reported from previously sequenced species (Stelzer 2011; Stelzer et al. 2011; Brown and Walsh 2019). Despite duplications and repetitive elements being hypothesized to contribute to increases in genome size (Gregory 2005; Lynch 2007), we did not find this to be the case among sequenced taxa (Fig. 1). Instead, we did observe higher numbers of predicted proteins with increases in genome size (Table 2). In addition to the number of predicted proteins, genome size was positively correlated with size-specific respiration rate (Table 4) as observed in previous studies (Hughes and Hughes 1995; Gregory 2005; Wright et al. 2014).

### Monogonont Genome Architecture

We used asexual clonal cultures to generate sufficient genetic material for ONT sequencing. Read coverages for assembled genomes ranged from 45 to 321 times the size of the assembly; sufficient for scaffold assemblies containing larger (>1 Mb) contiguous sequences. By examining the size of the largest contig, N50, L50, and duplications identified in the BUSCO analysis of the different assemblies, we can see variation within the assemblies, with some being more complete, e.g. *Hexarthra* sp., and others, such as *A. girodi*, where additional sequencing with new technologies could help to produce a more complete assembly. When comparing the assembly to flow cytometry genome size ratio, there appears to be no correspondence between the differences in percent coverage and complete BUSCO scores. This is exemplified by the genome size in *Hexarthra* sp. where coverage was 321× and the BUSCO completeness score was 96.5% but there was only a 84.8% correspondence in assembly:flow genome size ratio (Tables 2 and 3).

**Table 3:** Genome sizes for seven monogonont species estimated from whole-genome sequencing and flow cytometry

Species	Flow cytometry (pg)	Assembly results (pg)	Difference (pg)	Assembly/flow (%)
*Asplanchna girodi*	ND	0.144	ND	ND
*Euchlanis kingi*	0.173	0.126	−0.047	72.8
*Epiphanes brachionus*	0.096	0.103	0.007	107.3
*Epiphanes chihuahuaensis*	0.106	0.073	−0.033	68.9
*Lacinularia flosculosa* ^a^	0.133	0.143	0.010	107.5
*Notommata copeus* ^a^	ND	0.200	ND	ND
*Plationus patulus* ^a^	0.205	0.221	0.016	107.8
*Hexarthra* sp.^a^	0.046	0.039	−0.007	84.8
*Sinantherina socialis* ^a^	0.122	0.109	−0.013	89.3

Estimates using flow cytometry were not available for *A. girodi* or *N. copeus*.

ND, no data.

^a^Indicates that flow cytometry data are from Brown and Walsh (2019).

The role of TEs in contributing to genome size in rotifers was first investigated in bdelloid rotifers where it was found that they comprise ∼19% to 45% of the genome (Nowell et al. 2021). Blommaert et al. (2019) found substantial numbers of TEs in the genomes of various strains of *B. plicatilis* (14% to 25% repetitive elements) and *Brachionus calyciflorus* (39%). In the monogonont *B. asplanchnoidis*, Stelzer et al. (2021) found that the number of repetitive elements was consistent among genomes of closely related lineages and that the size and number of independently segregating genomic elements resulted in substantial increases in genome size (Blommaert and Stelzer 2022). We note that the ONT N50 scores were sufficiently long to resolve many individual repeat regions, permitting us to compare the number of repetitive elements across genomes. Thus, TEs represented ∼9% to 17% of the genomes, which was similar to other sequenced monogonont taxa. Our larger genomes fall within the previously reported range, and the smaller genomes fall just below the values obtained for *B. plicatilis* (Stelzer et al. 2011). Overall, the classes of elements were consistent across species, with two exceptions. First, there was an increase in the “other” group of elements found in both *Hexarthra* sp. and *N. copeus*. Second, there was a reduction of retroelements in *Hexarthra* sp. Combined, these studies indicate that TEs are prevalent in monogonont genomes but can vary in composition and prevalence.

Next, we identified a set of rotifer orthologous protein clusters shared among the species examined. This comprised 3,286 clusters with 46,093 proteins, likely representing conserved proteins present across taxonomic groups. Similarly, Hagemann et al. (2024) found a large set of shared proteins when comparing singular representatives from the major groups of rotifers (e.g. bdelloids, monogononts, and seisonids). These conserved sets are likely necessary for basic life functions, such as respiration, metabolism, and/or reproduction. Studies by Byeon et al. (2021, 2022) investigating orthologous genes in five brachionid species (*Brachionus angularis*, *B. calyciflorus*, *Brachionus rotundiformis*, *B. plicatilis*, and *Brachionus koreanus* [Hwang et al. 2014]) found that they shared ∼70% of orthologous genes. In our work, the closely related species *L. flosculosa* and *S. socialis* share similar lifestyle traits and a large portion of protein clusters (∼86%). Habitat also may play a role in the conservation of genes, as the marine species *P. similis* and *B. plicatilis* shared a large portion of their orthologous protein clusters (∼60%), potentially representing conserved proteins required to survive in saline environments. This is also seen in the gene contraction and expansion analysis of the gnesiotrochan rotifers, where the planktonic *Hexarthra* species has lost 770 protein clusters when compared to the genomes of the sessile species *L. flosculosa* and *S. socialis.* Furthermore, *L. flosculosa* and *S. socialis* genomes gained 195 clusters of related genes. Similarly, in our study, the two species of *Epiphanes* shared ∼84% of orthologous genes, but in both phylogenetic analyses, members of Epiphanidae (*E. brachionus* and *E. chihuahuaensis*) did not cluster together. This may be because of the small number of taxa sequenced from each genus, since they are clearly closely related based on morphological features of the buccal field, trophi (jaws), and placement of the foot (Koste and Shiel 1987). An alternative explanation is that the clustering represents shared protein clusters, not a phylogenetic analysis, and this could be driven by lifestyle, environment, or other factors. While additional annotated genomes are necessary to identify genes related to specific lifestyles or habitats, our contribution greatly expands the taxonomic breadth of genomes, which can be applied to these and other evolutionary innovations.

There are a variety of additional reasons for genome variation, both expansion and reduction, including large genomic region duplication events or smaller expansion of protein clusters. Using SyMAP to investigate self-synteny, no large duplication events were identified, suggesting that large regions of the genomes had not been duplicated in the past. Synteny between species was seen in some, but not all, taxa (data not shown). Similarly, when examining the 11 rotifer genomes, some synteny is detected but because of random fragmentation patterns seen in the scaffolds across species, it is currently infeasible to explore large-scale structural variation such as genome rearrangements. This would require alternative methods such as Illumina sequencing with Hi-C libraries.

### Genome Size and Monogonont Life Histories

Brown and Walsh (2019) found that genome size was significantly smaller in planktonic and solitary rotifers than in sessile and colonial species. This aligns well with the metabolic hypothesis of genome size and the energetic advantage hypothesis of coloniality (Wallace 1987). Under both hypotheses, larger genome sizes are expected in less energetically taxed systems, such as colonies, if they provide an energetic advantage or in sessile animals, which do not incur the cost of constant swimming. Respiration rates measured in this study are consistent with those previously reported for rotifers, ranging from 0.612 to 11.364 pm/min (Table 4; Galkovskaja 1987; Kirk et al. 1999). In other animals, smaller genome sizes are associated with higher metabolisms; for example, smaller genome sizes are found in birds with faster, more energetic flight (Wright et al. 2014). The metabolic theory of genome size assumes that genome size is being constrained by the organism's metabolism; if it is being constrained by another factor, genome size may no longer be negatively correlated with metabolism. We found that genome size and size-specific metabolic rate are positively correlated in rotifers, which suggests that the metabolic theory of genome size does not directly apply to them. Life cycle speed is known to be an important factor in constraining genome size in insects, which have the smallest genome sizes in high latitudes, where they also may have a lower metabolic rate due to temperature but require a fast life cycle due to the short growing season (Alfsnes et al. 2017). For example, whereas *Hexarthra* sp. are ∼12% larger, they have respiration rates ∼30% higher than *H. mira* (Kak and Rao 1998). Among the species we examined, the *Hexarthra* species has the fastest developmental time (Schröder et al. 2007), and its genome size being nearly equal to other *Hexarthra* species (Brown and Walsh 2019) suggests that these rotifers may be adapted for a higher metabolism related to their fast development. In cases where life cycle speed is under selection, adult metabolism may become uncoupled from genome size. The *Hexarthra* species we studied had the smallest genome and also has a novel life history (Schröder et al. 2007). However, their respiration rates are similar to other *Hexarthra* (Galkovskaja 1987). Conversely, *P. patulus* and *L. flosculosa* possessed large genomes (Table 2) and high metabolic rates (Table 4). We posit that this is due to the need to produce extensive mucilaginous structures, acting as anchoring threads in *P. patulus* or the gelatinous colony covering in *L. flosculosa*, that are likely considerable energetic investments.

**Table 4:** Characteristics of species included in the analysis of genome size

Species	Female body length without foot (µm)	Respiration rate (pmol/min)	Other features
*Asplanchna girodi*	565	0.851^a^	Freshwater, planktonic
*Euchlanis kingi*	210	0.973^a^	Freshwater, littoral
*Epiphanes brachionus*	231	2.258	Freshwater, littoral
*Epiphanes chihuahuaensis*	466	2.659	Freshwater, littoral
*Lacinularia flosculosa*	316	8.511^a^	Freshwater, colonial, sessile
*Notommata copeus*	1,081	ND	Freshwater, littoral
*Plationus patulus*	154	6.761^a^	Freshwater, planktonic
*Hexarthra* sp.	198	0.797^a^	Freshwater, planktonic
*Sinantherina socialis*	275	1.202^a^	Freshwater, colonial, sessile
*Proales similis*	88^c^	ND	Marine, planktonic
*Brachionus plicatilis*	170 to 320^b^	ND	Inland saline, brackish, marine, planktonic

ND, not determined using our methods.

^a^Allometric scaling of respiration rate with colony size (from Brown and Walsh 2024).

^b^From Snell et al. (2019).

^c^From Doohan (1973).

Environmental factors also may influence genome size. For instance, it has been hypothesized that genome miniaturization in tardigrades is related to selective factors associated with the meiofaunal habitats they occupy (Gross et al. 2019), constraining body size and cell size. We recovered genome sizes ranging from ∼38 to 216 Mb containing roughly ∼11,000 to 36,000 genes (Fig. 1; Table 2). Among these, we recovered the smallest genome for a *Hexarthra* species collected from an ephemeral rock pool (Table 2). We posit that the reduction in genome size is likely due to a compressed life cycle constrained by the shortened hydroperiod of their rock pool environment. Future research attempting to understand how lifestyle and niche space influence genome size would benefit from genome sampling of additional rotifer species representing a variety of locations, which may help elucidate patterns of genome size across geographic space and lifestyles. Rotifers may represent an ideal study system to test whether genome size may follow Bergmann's rule, which states that body size is larger in species from colder regions than those inhabiting warmer regions (Blackburn et al. 1999; Campbell et al. 2021). Aquatic insects appear to follow the rule inversely, but marine crustaceans follow the rule (Alfsnes et al. 2017; Campbell et al. 2021). The difference is thought to be related to shorter growing seasons further north for aquatic insects but longer and slower development overall in cold marine waters. We hypothesize that rotifers of inland waters that experience similar seasonal pressures to aquatic insects are expected to follow the same pattern (inverse Bergmann's rule), whereas rotifers inhabiting short hydroperiod habitats may follow Bergmann's rule.

### Other Considerations and Conclusions

Cryptic species are well known and widespread in both monogonont (Gómez et al. 2002; Schröder and Walsh 2007; Walsh et al. 2009; García-Morales and Elías-Gutiérrez 2013; Leasi et al. 2013; Obertegger et al. 2014; Kimpel et al. 2015; Kordbacheh et al. 2017; Michaloudi et al. 2017; Mills et al. 2017; García-Morales and Domínguez-Domínguez 2019; García-Morales et al. 2021) and bdelloid rotifers (Fontaneto et al. 2007, 2008, 2009, 2011; Birky et al. 2011; Cakil et al. 2021). Importantly, cryptic species may possess different genome sizes (Stelzer et al. 2011), and thus, genome size may be an appropriate measure of taxonomic diversity for Rotifera. We acknowledge that genome size may not be appropriate for all species’ taxonomy. For example, genome size evolution driven by inheritable small chromosomal elements in *B. asplanchnoidis* makes genome size alone less valuable for taxonomy since genome size does not appear to be species specific and evolving rapidly (Stelzer et al. 2021). Thus, when possible, incorporating karyotyping along with genome sequencing and flow cytometry would be best. Doing so can reveal further insight into the number of chromosomes and their arrangement, as well as ploidy levels in rotifers as several species are known to have increased genome size via ploidy (Walsh and Zhang 1992; Mark Welch et al. 2008; Hur et al. 2009; Stelzer et al. 2021).

Rotifers are a diverse group of aquatic microorganisms that are equally diverse in the size and content of their genomes. The phylogenetic breadth explored here extends the base of our knowledge of rotifer genomes but also highlights gaps in our understanding of genomic features. As sequencing of both DNA and RNA becomes less expensive and more reliable, our ability to create rotifer-specific models to predict genes, repetitive elements, duplication events, and/or genome reduction will be made possible. For each of the species sequenced, a more refined genome assembly could be accomplished but alternative sequencing techniques (e.g. Illumina Hi-C reads, direct ONT sequencing of larger [>20 kb] genomic fragments) may be needed. Future studies also should determine the feasibility of producing information on epigenetic maps and pangenomes across monogonont rotifers to better determine genetic versus environmental responses to environmental conditions across generations. In addition, as more genomes are made available, lifestyle features can further be linked to variations in size and genomic content.

## Materials and Methods

### Collection, Culture, and DNA Extraction

Species from various rotifer families were collected opportunistically from freshwater habitats in the United States of America or hatched from sediments from Ryan's 2 billabong, Australia (i.e. *S. socialis*) (Table 5). Asexual cultures were established to minimize the occurrence of genome variation; however, sexual reproduction may have occurred occasionally in some of the long-term cultures. All species were cultured in modified MBL medium (Stemberger 1981) and fed a mixture of *Chlamydomonas reinhardtii* (UTEX Culture Collection of Algae at UT-Austin [UTEX] strain 90), *Chlorella vulgaris* (UTEX strain 30), and *Cryptomonas erosa*. Rotifers (1,000 to 1,500) were serially washed nine times to remove food and debris before DNA extraction using the gMax Mini Kit (IBI Scientific). DNA extraction followed the manufacturer's solid tissue protocol with the following modifications: live rotifers were chilled and pelleted before the addition of the GST Buffer and Proteinase K. In the final step, 40 µl of elution buffer was used. DNA was quantified using QUBIT.

**Table 5:** Collection localities of species included in this study

Family/species	Location	GPS coordinates
**Asplanchnidae**		
*Asplanchna girodi*	La Mancha Wetlands, Doña Ana Co., NM	32.2780, −106.8286
**Brachionidae**		
*Plationus patulus*	Rio Grande, El Paso Co., TX	31.4302, −106.1422
**Epiphanidae**		
*Epiphanes brachionus*	Storm Drain, El Paso Co., TX	31.4302, −106.1422
*Epiphanes chihuahuaensis*	North Temp rock pool, HTSPHS, El Paso Co., TX	31.9246, −106.0424
**Euchlanidae**		
*Euchlanis kingi*	Crystal Lake, Belknap Co., NH	43.4443, −71.3118
**Hexarthridae**		
*Hexarthra* sp.	Tip Top rock pool, HTSPHS, El Paso Co.,TX	31.5447, −106.0245
**Flosculariidae**		
*Lacinularia flosculosa*	Laguna Prieta playa, HTSPHS, El Paso, Co., TX	32.9246, −106.0471
*Sinantherina socialis*	Ryan's 2 billabong, Australia	36.11072, 146.9666
**Notommatidae**		
*Notommata copeus*	Rattlesnake Springs, CAVE, Eddy Co., NM	32.1095, −104.4717

CAVE, Carlsbad Caverns National Park, Eddy Co., NM; HTSPHS, Hueco Tanks State Park and Historic Site, El Paso Co., TX.

### Flow Cytometry

We followed the methods of Brown and Walsh (2019) to estimate genome size for *E. brachionus* and *E. chihuahuaensis*. For the remaining taxa, we used values from Brown and Walsh (2019). Briefly, 500 to 1500 individuals of each species were prepared for cytometry by grinding them with 15 strokes of a Dounce tissue homogenizer on ice. Then, samples were digested with trypsin (0.003%), cleared of RNA and trypsin (RNase A 0.01% and trypsin inhibitor 0.05%, respectively), and stained with propidium iodide (0.04%) overnight. These solutions were all dissolved in stock a buffer consisting of 0.4 mM trisodium citrate dihydrate, IGEPAL at 0.1% *v*/*v*, 1.5 mM spermine tetrahydrochloride, and 0.5 mM Tris(hydroxymethyl)aminomethane at pH 7.6. To serve as a reference standard for genome size, we used *Drosophila melanogaster* Canton S (0.18 pg) and *Caenorhabditis elegans* N2 (0.10 pg) (Carolina Biological Supply, Burlington, NC, USA). Fly heads (1 to 2) and nematodes (∼1,000) were processed with rotifers. Stained samples were run through a Gallios flow cytometer at the Border Biomedical Imaging core facility at the University of Texas at El Paso. The relative fluorescence of propidium iodide of the reference (*D. melanogaster* and *C. elegans*) versus the sample (rotifer) nuclei was used to estimate genome size. We tested for correlation between genome sizes determined by flow cytometry and genome assembly.

### Low-Input Oxford Nanopore Library Preparation and Sequencing

Oxford Nanopore (ONT) library preparation followed protocols outlined in the low input by PCR Ligation Sequencing 1D kit (i.e. SQK-LSK110; Oxford Nanopore). In short, PCR amplification followed manufacturer's protocols, except that we used AccuStart Long Range SuperMix (QuantaBio, MA, USA) instead of the suggested master mix (see ONT protocol). Our PCR protocols followed an initial denaturation at 95 °C for 3 min and 18 cycles of denaturation at 95 °C for 15 s, primer annealing at 62 °C for 15 s, and extension at 65 °C for 17 min, with a final extension at 65 °C for 17 min before holding temperate at 4 °C. Library preparation proceeded following protocols in subsequent steps. Note that we used a 1× concentration of sparQ PureMag Beads (QuantaBio, MA, USA) for all cleanup steps. We conducted double-stranded DNA quantification after each cleanup step on a Qubit 3.0 and using High Sensitivity assays per ONT protocols. We loaded total library concentrations ranging from 800 ng to 1 µg on a single FLO-MIN106D flow cell, with sequencing occurring on an ONT MK1C sequencer. Only one rotifer genome was sequenced on each flow cell, and we ran each flow cell until no pores were available for sequencing with pore mixing occurring every hour.

### Assembly and Polishing

For each genome, the reads were assembled using the Flye assembler (Kolmogorov et al. 2019). The assembly was further refined by performing three rounds of polishing using Minimap (Li 2016) and Racon (Vaser et al. 2017). The resulting contigs were sorted by size and any contig below 1,000 bases was removed. A BLAST search against the *nt* database was used to identify contigs that were likely contaminants (e.g. food sources, human, and fungi). Contigs that had a match to a contaminant with an *e*-value of 1 × 10^−5^ and the match was more than 50% of the length were removed. A similar self-BLAST was conducted to remove potential contigs resulting in misassemblies with the same cutoff as used for contaminants. The assembly quality was checked using both BUSCO (Seppey et al. 2019) and Quast v. 5* (Mikheenko et al. 2018).

### Genome Annotation

Protein coding regions of the assembled genomes were predicted using Braker2 (Stanke et al. 2006; Stanke et al. 2008; Buchfink et al. 2015; Hoff et al. 2016, 2019; Bruna et al. 2021). The functional annotation of the resulting protein sequences was done in the functional annotation package of OmicsBox (www.biobam.com). BLASTP (version 2.13; Altschul et al. 1990) was used first to search the *nr* database locally to identify the top 20 protein matches. InterProScan (Jones et al. 2014) was run within OmicsBox to identify protein families for each sequence. Using the results of the BLAST and InterProScan, gene ontology terms and Enzyme Commission numbers were then assigned to the predicted proteins. The summarized files containing the functional information were then exported in both a tab-delimited table and wego formatted file. Assemblies of *B. plicatilis* and *P. similis* were downloaded, and the functional annotation process was repeated in the same manner for comparison.

### Genome and Protein Comparisons

Using RepeatMasker (Smit et al. 2013–2015), TEs and other repetitive elements were identified at the Metazoan level due to the lack of availability of a Rotifera-specific dataset. The relationship between repetitive and nonrepetitive DNA content per genome was investigated by linear regression. Synteny maps to align the assemblies were constructed using SyMAP (Soderlund et al. 2011). These were used to check for large genomic duplication events. BLAST searches were also used to investigate regions of duplication within each genome. OrthoVenn3 (Sun et al. 2023) was used to compare the proteins identified by Braker2 in each of the genomes.

### Metabolic Rate Determination

Oxygen respiration rate was used as a proxy for rotifer metabolism as in Brown and Walsh (2024). Briefly, rotifers were prepared for experiments by rinsing them in MBL medium and removing debris to clean them. Oxygen respiration rate was measured using a microplate reader and microplate sensor system (SDR SensorDish Reader; Loligo Systems). The microplate was calibrated prior to each run with a two-point calibration, using 1% m/v sodium sulfite in MBL medium and aerated MBL medium as 0% and 100% oxygen standards, respectively. To measure oxygen consumption, rotifers were added to up to 18 of the 80-µl wells, with the remaining wells containing only aerated MBL medium to function as controls. Measurements were taken for a 2-h period at 20 °C in the early afternoon. Oxygen consumption rate was estimated using the program MicroResp (Loligo Systems) by subtracting the blanks from the values, excluding the first 5 min of each run (temperature acclimation of system in incubator) and replicates with *R*^2^ < 0.5. Data for some species were taken from Brown and Walsh (2024) (see Table 4).

### Size-Specific Metabolic Rate and Genome Size

Genome-size was compared to size-specific metabolic rate using mixed-effect linear regression with hierarchical taxonomic ranks as mixed effects. Rotifer sizes used were maximum lorica lengths obtained from the literature (Stemberger and Gilbert 1985; Kak and Rao 1998) or body lengths (excluding the foot) as measured from photographed animals (average of at least ten individuals) using ImageJ (Rasband 1997-2018; Schneider et al. 2012). Exclusion of the foot allows for more accurate comparison of rotifer sizes as *Asplanchna* and *Hexarthra* lack a foot. Body lengths for *B. plicatilis* and *P. similis* were obtained from the literature (Snell et al. 2019) as we did not have these species in culture. Analyses were conducted using the R-packages lme4, lmerTest, and MuMIn (Bates et al. 2015; Kuznetsova et al. 2017; Bartoń 2022).

## Supplementary Material

evaf041_Supplementary_Data

## Data Availability

Sequencing reads are available through NCBI's Sequence Read Archive. Assembled genomes are available through NCBI. All other files are available at the UTEP Bioinformatics Repository (https://datarepo.bioinformatics.utep.edu/getdata?acc=GSYH6KK3OV0Q32M).
