# Supplementary material for: Comparing Small and Large Genomes Within Monogonont Rotifers
Source: Genome Biol Evol. 2025 Mar 6;17(3):evaf041. doi: 10.1093/gbe/evaf041 (PMC11954553; doi:10.1093/gbe/evaf041)
Supplement: evaf041_Supplementary_Data [file evaf041_supplementary_data.zip › SD3.pdf]

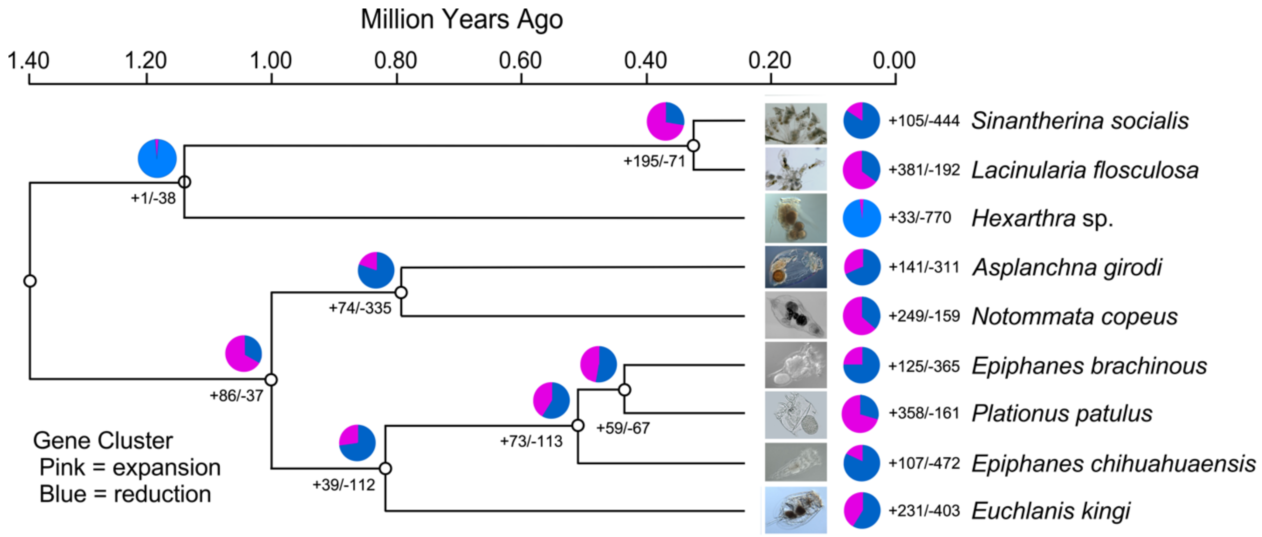

Supplemental Document 3: OrthoVenn3 neighboring joining phylogenetic analysis of selected rotifers based on whole genome sequences showing gain/loss of protein clusters at the nodes. Pie charts highlight the increase (pink color) or decrease (blue color) of protein clusters at each split of a branch. Images of the rotifers are found at the terminal nodes of the tree.
