# Supplementary material for: Comparing Small and Large Genomes Within Monogonont Rotifers
Source: Genome Biol Evol. 2025 Mar 6;17(3):evaf041. doi: 10.1093/gbe/evaf041 (PMC11954553; doi:10.1093/gbe/evaf041)

Supplemental Document 2: OrthoVenn3 output highlighting overlap of predicted protein clusters in the rotifer genomes. Dark green ovals mark which rotifer species are present in the overlap while lighter green ovals shows absence of the species within the row. The total clusters across the selected species is shown next to the total protein count. The frequency graph at the end of the row highlights the percent of the total proteins that specific for each of the species. All colors are consistent to their respective species throughout the figure.

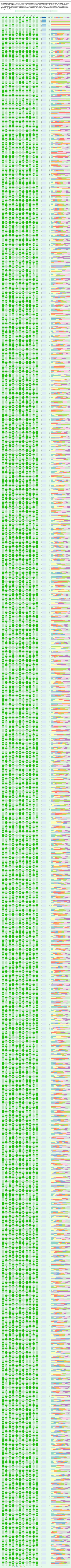

Supplement: evaf041_Supplementary_Data [file SD2.pdf › SD2.pdf]
